# Very low rates of screening for metabolic syndrome among patients with severe mental illness in Durban, South Africa

**DOI:** 10.1186/s12888-014-0228-5

**Published:** 2014-08-12

**Authors:** Shamima Saloojee, Jonathan K Burns, Ayesha A Motala

**Affiliations:** Department of Psychiatry, Nelson R Mandela School of Medicine, University of KwaZulu – Natal, Private bag X3, Congella, 4013 Durban, South Africa; Department of Diabetes and Endocrinology, Nelson R Mandela School of Medicine, University of KwaZulu - Natal, Durban, South Africa

**Keywords:** Metabolic syndrome, Screening, Severe mental illness, Antipsychotics

## Abstract

**Background:**

Sub Saharan African is experiencing the largest increase in the prevalence of type 2 diabetes mellitus and cardiovascular disease globally. Metabolic syndrome (MetS) is a cluster of risk factors for these conditions. There is a consistently higher prevalence of cardiometabolic disease among individuals with severe mental illness (SMI) compared to the general population worldwide .However, it is known from research in high income countries that screening for MetS in patients with SMI is low. The objective of this study was to document the extent of the expected low frequency of testing for all the components of the metabolic syndrome (MetS) in patients with SMI in a low middle income country.

**Methods:**

This was a cross sectional study, undertaken from January to June 2012 on out-patients with SMI who were treated with antipsychotic medication for at least 6 months. The study measured the proportion of participants who were tested for MetS in the previous year.

**Results:**

The study included 331 (M: F; 167:164) participants with a mean age of 35.2 ± 11.98 years. The majority (78.8%) were black South Africans. Only 2 subjects (0.6%) were screened for all five components of MetS. Regarding the individual components, 99%, 0.6%, 3.9% and 1.8% were screened for raised blood pressure, abdominal obesity, hyperglycaemia, hypertriglyceridaemia and decreased high density lipoprotein cholesterol respectively.

**Conclusion:**

It is unacceptable that less than one percent of our participants were adequately screened for modifiable risk factors for type 2 diabetes mellitus and cardiovascular disease which are the most common causes of mortality among patients with SMI. These results highlight the need for translating guidelines into action in low and middle income countries.

## Background

Worldwide, individuals with severe mental illnesses (SMI) such as Schizophrenia and related disorders, have a consistently higher prevalence of metabolic syndrome (MetS) than the general population [1,2]. Severe mental illness (SMI) refers to mental illnesses which are persistent and cause substantial social and/or occupational dysfunction [3]. MetS is a cluster of risk factors for cardiovascular disease (CVD) and type 2diabetes. According to the Joint Interim Statement (JIS) criteria any three of the following five components are necessary for the diagnosis of MetS: increased waist circumference, raised blood pressure, increased triglycerides, decreased high density lipoprotein (HDL) cholesterol and raised plasma glucose [4].

The increased prevalence of MetS in individuals with SMI has been attributed to several factors including the mental illness itself, the adverse effects of antipsychotic medication and traditional risk factors for CVD and type 2 diabetes mellitus such as obesity, unhealthy high fat low fibre diets, physical inactivity and smoking, which are common in patients with SMI [5].

Antipsychotics are an important cardiometabolic risk factor for MetS with a variable risk for MetS among them [6,7]. A meta-analysis comparing the metabolic side effects of second generation antipsychotics (SGAs) showed that olanzapine and clozapine were associated with the highest risk for elevations in weight, cholesterol and glucose [7]. The metabolic risk associated with SGAs led to United States Food and Drug Administration (FDA) black box warnings and the development of several international treatment guidelines for routine metabolic screening and monitoring in patients taking SGAs [8]. Although the evidence for the metabolic side effects of first generation antipsychotics (FGAs) is not as robust as that for SGAs, there is also evidence in the early literature to confirm the metabolic risk profile of FGAs [9].

The extent and frequency of screening is debatable, but there is global consensus that baseline metabolic screening should form part of the standard of care for all patients with a regular prescription of an antipsychotic [1,2,5-8].

However, reports of metabolic screening practices in large cohorts of patients from high income countries (HIC) have shown that metabolic testing remains inadequate despite widespread dissemination of the guidelines and a high level of awareness of the metabolic risks associated with SGA medications among psychiatrists [10-12]. A meta-analysis of screening practices from five HIC countries (United States of America (USA), United Kingdom (UK), Australia, Canada and Spain) found that routine baseline screening was above 50% for blood pressure and triglycerides only and that glucose and cholesterol were measured in less than 50% of individuals with mental illness. No data from low middle income countries (LMIC) was included in this meta-analysis [10].

The 2005 South African guidelines for managing metabolic abnormalities in patients with schizophrenia recommended monitoring anthropometric measurements, plasma glucose and serum lipids prior to initiation of SGA medication, and at 6 weeks, four months and twelve months thereafter [13].

Despite these recommendations, a study from the Western Cape in South Africa showed that in outpatients with SMI, no patient had a waist circumference measurement. In that study, anthropometric measures were examined without blood tests [14]. Marsay and Szabo also found that four months after initiation of Olanzapine there was so little screening that data interpretation had no value [15].

This study was motivated by the dearth of information on the frequency of monitoring for all the components of the MetS in patients with a SMI nationally as well as the general paucity of information from LMIC. It is not known whether the frequency of metabolic testing for all the components of MetS in patients with a SMI in South Africa is similar to those observed in European and North American countries. Even less is known about medical care for patients with a SMI who have no public or private health insurance.

The aim of this study therefore, was to determine the frequency of testing for all the components of MetS in patients attending a general hospital psychiatric unit in Durban, South Africa.

## Methods

### Design and sample

This was a cross sectional study, undertaken from January to June 2012 in outpatients with SMI, aged 18–65 years who were treated with antipsychotics for at least 6 months and attending the psychiatric unit at King Edward VIII Hospital in Durban, KwaZulu - Natal South Africa.

This public health facility is the second largest hospital in the Southern hemisphere and is the main teaching hospital of the Nelson R Mandela School of Medicine. The hospital serves predominantly uninsured patients from disadvantaged backgrounds. KwaZulu - Natal province has the highest prevalence of HIV disease in the country, resulting in a high burden of psychiatric disorders associated with HIV [16]. The hospital’s psychiatric outpatient clinic provides care and treatment at both primary and specialised levels because community - based mental health services in the district are under-developed and poorly funded. The clinic serves as a primary mental health care clinic for individuals residing in close proximity to the hospital because the local clinic is overcrowded and there is frequently a shortage of SGAs. Psychiatric registrars run the clinic under the supervision of specialist psychiatrists.

In this study the following were defined as SMI: schizophrenia, bipolar 1 mood disorder, major depressive disorder with psychotic features, psychotic or mood disorder associated with a general medical condition and schizoaffective disorder. The daily outpatient attendance registers in the unit were used to identify adult patients aged 18–65 years with a continuous prescription of an antipsychotic for at least six months.

### Data collection

All consenting participants provided information for a questionnaire including information regarding age, gender, ethnicity, employment status, cigarette smoking, current or past diagnosis of hypertension, diabetes and HIV disease. Available information on waist circumference, blood pressure, weight, cholesterol and glucose testing in the preceding year was recorded. Documentation in the psychiatric case notes of testing for any of the Mets components (blood pressure, waist circumference fasting blood glucose and lipids) was considered to be evidence that testing took place. We did not search the hospital laboratories’ database for glucose and lipid testing. Participants’ records were also examined to obtain the diagnosis of mental illness and the antipsychotic prescribed. Compulsory hospital audits have ensured that the psychiatric case records are legible and contained the information required for this study. Psychiatric records are filed in the psychiatric clinic and not in the general hospital filing system. The records are thus readily available.

### Measures

The study measured the proportion (%) of participants who were tested for the components of MetS in the previous year.

### Statistical analysis

Statistical analysis was undertaken using the Statistical Package for Social Sciences (SPSS version 19). Means and standard deviations were computed for continuous variables and percentages or proportions for categorical variables. Descriptive statistics (percentages or proportions) were used to report the results. Participants who had fasting glucose or lipid test results and who were taking olanzapine or clozapine were compared to participants taking other antipsychotics using chi-squared tests.

This study was approved by the Biomedical Research Ethics Committee of the University of KwaZulu - Natal.

## Results

This analysis included 331 participants with SMI who were treated with an antipsychotic for at least six months. Demographic and clinical characteristics of the participants are shown in Table 1. The gender distribution was equal. Most participants were of black African ancestry (78.8%). The mean age of the study subjects was 35.2 ± 11.98 years and 40% of the participants were in the 18–30 year age group. Smoking, a modifiable risk factor for cardiovascular disease was identified in 23.3% (n = 77) of the participants and 20.2% were HIV positive.

**Table 1:** **Demographic and clinical characteristics of study participants (n = 331)**

**Clinical characteristics**	
Gender n (%)	
Male	167 (50.5%)
Female	164 (49.5%)
Ethnicity n (%)	
African	261 (78.8%)
Indian	28 (8.5%)
White	32 (9.7%)
Coloured	10 (3%)
Age (years) mean ± SD	35.2 ± 11.98
Age distribution n (%)	
18-30 years	141 (42.6%)
31-40 years	82 (24.8%)
41-50 years	60 (18.1%)
51-64 years	48 (14.5%)
Employment n (%)	
Unemployed	200 (60.4%)
Formal	32 (9.7%)
Student	36 (10.9%)
Disability grant/old age pension	63 (19%)
Diagnosis n (%)	
Schizophrenia	163 (49.3%)
Major Depressive Disorder	15 (4.5%)
Bipolar Mood Disorder	49 (14.8%)
Schizoaffective Disorder	36 (10.9%)
Mood/psychotic disorder due to a general medical condition	68 (20.5%)
Antipsychotic- medications n (%)	
Risperidone	125(37.8%)
Chlorpromazine	37 (11.2%)
Haloperidol	54 (16.3%)
Zuclopenthixol Decanoate	19 (5.8%)
Clozapine	15 (4.5%)
Olanzapine	13 (3.9%)
Quetiapine	45 (13.6%)
Amisulpride	12 (3.6%)
Aripiprazole	11 (3.3%)
History of [n (%)]:	
Smoking	77 (23.3%)
HIV disease	67 (20.2%)
Hypertension	12 (3.6%)
Type 2 diabetes	5 (1.5%)

Results expressed as n (%) or mean ± SD.

Three out of every four participants were either unemployed or on state disability grants. Just over two thirds of the group were treated with a SGA. The most and least commonly prescribed antipsychotics were risperidone (38%) and aripiprazole (3%), respectively. Clozapine was prescribed for 15 (4.5%) and olanzapine for 13 (3.9%) of the 331 participants.

Of the 331 participants only 2 (0.6%) were screened for all five components of MetS (Table 2). The most frequently monitored component of MetS was blood pressure (99%) and the least frequent was waist circumference (0.6%).The majority had a random plasma glucose test record in the last year (96.6%) but fasting glucose testing was infrequent*.* Participants who had a case note record of fasting glucose (olanzapine 53.8%, clozapine 15.4%, risperidone 15.4%, chlorpromazine 7.7%, haloperidol 7.7%, p < 0.001) or lipids (olanzapine 33.3%, clozapine 50%, risperidone 16.7%, p < 0.001) were significantly more likely to be taking clozapine or olanzapine. However, of all participants taking olanzapine or clozapine only 8% and 7% respectively had psychiatric case note records of both fasting glucose and lipid tests.

**Table 2:** **Rates of screening forMetS components in the past year in study participants (n =331)**

**MetS Component**	**Screened n (%)**
Blood Pressure	328 (99%)
Fasting blood glucose (Random blood glucose)	13 (3.9%) 320 (96.6%)
Fasting serum lipids	6 (1.8%)
Waist circumference	2 (0.6%)
All components	2 (0.6%)

Forty two per cent (n = 138) of patients with SMI relied on the hospital’s psychiatric clinic for their general medical care; only 10.5% visited their general practitioner for medically related illnesses (Table 3).

**Table 3:** **Treatment settings for general medical care for study participants (n = 331)**

**Site**	**n (%)**
General Practitioner	35 (10.6%)
Psychiatric clinic	138 (41.7%)
Other clinic e.g. medical/surgical follow up clinic in the hospital	121 (36.5%)
Local Primary/Community health clinic	37 (11.2%)

Stratification of testing by age and site of general medical care did not show any differences in the monitoring of older versus younger patients and patients who relied on the psychiatric clinic for their physical health care compared to those who did not*.*

## Discussion

This study found very low rates of screening for all the components of MetS in patients with SMI treated with antipsychotics from a LMIC; moreover except for blood pressure, screening for the other components of MetS was unacceptably low. The finding that only 0.6% of participants were screened for all the components of MetS is to the best of our knowledge, the lowest recorded in the literature. Direct comparisons with other regions are not reliable because of the substantial heterogeneity in the study settings, samples, within country differences, time frames for screening and selective reporting of individual MetS components in the literature. For example, only eight of the 48 studies in the meta-analysis by Mitchell et al. [10] reported on fasting samples. Nevertheless, data from HIC are presented to contextualise our findings.

High rates of metabolic screening have been reported in Veterans Affairs (VA) settings in the USA [17-19] and post quality improvement programmes implementation in Spain [20] and the UK [21]. In a VA setting, Khatana et al. [17] found that about 80% of patients with a SMI were monitored for all components of the MetS and similarly Shi and colleagues [18] report that 75.8% of patients with schizophrenia received glucose and lipid monitoring using the Veterans Integrated Service Network (VISN) 16 electronic records of the Veterans Health Administration. In contrast, rates of below 30% for glucose and 15% for lipids have been reported among US Medicaid [22] and commercial insurance enrollees [23]. A likely explanation for this discrepancy is the presence of a primary care clinic which is co-located and integrated into the outpatient mental health clinic at VA medical centres, the older age of the participants in the VA system and their more effective metabolic monitoring programmes. Bobes’ group [20] showed that waist circumference, lipid and glucose profiles were recorded in 33.2%,72.5% and 73.2% respectively of clinical charts following the dissemination of the Spanish consensus on physical health in patients with schizophrenia. In the UK, Gonzales et al. [21] report that glucose and lipids were monitored in 72.6% and 52.8% of patients respectively following a monitoring prompt and educational intervention programme. However, Mackin et al. [24] found that 0% of patients had a record of waist circumference and less than 10% had a blood glucose or lipid result recorded in a study from the Northeast of England. Similarly an Australian audit of discharged patients with schizophrenia found that only 3% had a post prandial blood sugar and 7.5% triglycerides and cholesterol recorded in their case notes. Waist circumference was not recorded in this study [25].

Therefore, our findings indicate local screening rates that are substantially lower than the general finding of suboptimal metabolic monitoring for patients taking antipsychotics from HIC [10]. We reported on fasting laboratory samples, included an overall rate of screening for all components of MetS and excluded weight or BMI as a measure of central adiposity which could have contributed to our low frequency of screening.

It is disappointing that only 3.9% of the participants had fasting blood glucose and 1.8% had fasting lipid (triglycerides, HDL cholesterol) tests given that both glucose and lipid abnormalities can occur without weight gain [26]. Furthermore, in spite of previous reports that clozapine and olanzapine are associated with substantially greater metabolic side effects than other SGAs less than 10% of the patients treated with clozapine or olanzapine in this study had both glucose and lipid tests [7]. In keeping with the study by Gul [27] there was a discrepancy between the frequencies of fasting and non - fasting blood tests in patients with mental illness. A likely explanation is that it is not routine for patients to be asked to attend follow up appointments in a fasting state and also most patients could not afford to return to hospital for repeat fasting blood tests.

More than three quarters (79%) of our participants were either unemployed or on a state disability grant.Previous studies have demonstrated that there is an inverse relationship between socioeconomic class and the prevalence of MetS [28]. Poor access to information and little understanding of the impact of lifestyle modification on cardiovascular risk has been shown to be associated with poverty and illiteracy among the general population of India [29]. The low frequency of metabolic monitoring in the current study may be partially attributable to the inadequate health education received by the patients who were mostly unemployed and with no health insurance.

Our findings also highlight some barriers to screening such as the lack of integrated mental and medical services locally and the poorly developed community – based psychiatric services resulting in an over reliance on institutional care. Many patients relied on the hospital’s psychiatric clinic for their general medical care. Poorer access to medical care for patients with a mental illness compared to patients without a mental illness has been documented in other settings [30]. The availability of medical services within the psychiatric clinic as well as dedicated metabolic monitoring clinics is associated with higher monitoring rates [17,31]. The South African public health care system is currently struggling to cope with very high case loads and patients experience long waiting times before being attended to. A fee, which is dependent on the income of an individual, is levied for attending the hospital but patients who cannot pay are not turned away. There is a laboratory located within the hospital which is part of the national health laboratory service. The institution is liable for the cost of services rendered by the laboratory. Individual patients do not pay for laboratory testing but doctors at our hospital are encouraged to order tests based on clinical indication and where the results of testing impact on further management. The attending doctor is responsible for all physical examinations in the psychiatric clinic. Although phlebotomists are employed throughout the hospital, a phlebotomist is not allocated to the psychiatric clinic and doctors are responsible for the drawing of bloods from the patients they attend to. A possible solution to improve our monitoring rates may be to introduce a model of shared care. A nurse – run metabolic monitoring programme has proved to be successful in the United States [32]. Mental health providers in LMIC need to extend their roles beyond taking care of the mental health of their patients and assume some responsibility for the physical health of their patients. Identification of patients at risk for MetS through screening should take place at the point of care for the mental illness.

Patients with a SMI have a 2–3 fold higher prevalence of type 2diabetes and CVD [1]. Smoking, a modifiable risk factor for CVD was identified in one in five of the participants in this study. A recent study showed that all CVD risk factors except for cholesterol were higher in patients with SMI than in the general population of the United States [33]. Furthermore, Lozano et al. [34] report that, although global disability and deaths due to diabetes and CVD are projected to increase by 2020, the largest increases in deaths are predicted for Sub-Saharan Africa (SSA). SSA is experiencing one of the most rapid rates of urbanization worldwide which may be contributing to a rising prevalence of non- communicable diseases (NCDs) in this region [35]. Against this background of a surge in the prevalence of NCDs in the general population, the mentally ill in SSA form part of a particularly high risk group for future CVD and diabetes mellitus. Our main concern therefore, is that because of the inadequate screening received by the overwhelming majority of patients in this study the associated risks will, in all likelihood go undetected.

This study has several limitations. The study was conducted in one general hospital and the generalizability of our results may be questionable. Our results are dependent on the accuracy of the information contained in the psychiatric case notes. It is likely that individuals may have been screened by health care providers in this or other hospitals in the district, but, that these results were not communicated to the attending psychiatrist.

## Conclusions

Our findings are clinically significant although they were not entirely unexpected. The significance of our study is that it quantifies the extent of the under-recognition of modifiable risk factors for CVD such as central obesity, hypertension, dyslipidemia, and hyperglycemia among patients with SMI. This study emphasises the lack of translation of guidelines to action for the prevention, early detection and treatment of these risk factors in a particularly high risk group of individuals. It highlights the importance of documenting the frequency of screening because this will hopefully support the allocation of resources for improved screening in LMIC.

## Abbreviations

MetS: Metabolic syndrome
SMI: Severe mental illness
SGAs: Second generation antipsychotics
FGAs: First generation antipsychotics
LMIC: Low-Middle income country
HIC: High income country
USA: United States of America
UK: United Kingdom
VA: Veterans affairs
